# Drivers and Consequences of Narrative Transportation: Understanding the Role of Stories and Domain‐Specific Skills in Improving Radically New Products†

**DOI:** 10.1111/jpim.12329

**Published:** 2016-08-01

**Authors:** Fiona Schweitzer, Ellis A. Van den Hende

## Abstract

This article investigates the role of transportation in concept tests (i.e., a vivid mental image of a new product concept and the way of using it) for radically new products. Based on transportation literature, the article proposes that concept descriptions in a story format can stimulate transportation. Further, the article builds on the literature on domain‐specific skills to propose that technological reflectiveness (i.e., the ability to think about the impact of a technological product on its users and society in general) and product expertise increase transportation. The article explores the effect that transportation has on the ability of consumers to enumerate the advantages and disadvantages of a radically new product and on their ability to provide valuable concept improvement ideas (i.e., ideas that are highly novel, feasible, and beneficial for consumers). A quasi‐experiment with 253 participants demonstrates that a story format, product experience with related product categories, and technological reflectiveness increased transportation with regard to radically new products. The empirical research also showed that transportation facilitates the enumeration of the advantages and the disadvantages of a concept, resulting in more valuable concept improvement ideas. These findings suggest that innovation managers should strive to evoke transportation in concept tests for radically new products, as transportation allows consumers to provide more valuable input.

## Practitioner Points

- When taking into account the kind of concept description and selection of customers, customers can provide valuable concept improvement ideas for radically new products
- Concept descriptions in a story format facilitate customers’ ability to provide such ideas
- More technologically reflective individuals are in a better position to come up with valuable concept improvement ideas for radically new products

## Introduction

Seeking help from consumers has become common practice in new product development (NPD). Common methods include online co‐creation challenges in which firms seek ideas to solve innovation problems. More specifically, in the ideation stage of the NPD process, this consumer input can outperform expert input in terms of originality and consumer value (Poetz and Schreier, 2012). Firms can start an NPD project with novel ideas. However, once these novel product ideas have become concepts, new consumer input is required. In concept tests, companies typically look for input for developing and improving concepts (Crawford and Di Benedetto, 2008; Peng and Finn, 2008; Peng, Li, and Wan, 2012).

Such input is easier to gather for incrementally new product (INP) concepts than for radically new product (RNP) concepts (Hoeffler, 2013). An RNP typically features new technology, offers new benefits, and requires new usage patterns (Veryzer, 1998). A lack of prior experience with an RNP makes it difficult for consumers to visualize RNP concepts (Hamel and Prahalad, 1994; Knudsen, 2007; O'Connor and Veryzer, 2001). Concept tests often confront consumers with unfinished products in the form of verbal statements both with and without graphical representations (Foley, 2012; Page and Rosenbaum, 1992; Peng and Finn, 2008). These concepts present the intended features of the final product. Consumers cannot try out the features to learn how to use the product, but instead have to imagine what the final product will look like and how they will potentially interact with it (Crawford, 1991; Hoeffler, 2003; Zhao, Hoeffler, and Dahl, 2012). Mentally visualizing the future usage of such a product is a feasible task for consumers if the presented concept is incremental, because it is then similar to products with which users are already familiar, enabling them to draw on their prior use experience. Consumers lack this familiarity with RNPs and cannot draw on prior use experience in order to understand RNPs and the potential advantages and disadvantages of their usage (Hamel and Prahalad, 1994; O'Connor and Veryzer, 2001; Veryzer, 1998).

The difficulties that a customer has in imagining the future usage of a product in a concept test reduce the customer's ability to provide information that helps managers improve RNP concepts (Hoeffler, 2003; Veryzer, 1998). At the same time, managers need this customer information to reduce market uncertainties and avoid market failure of the RNP (Callahan and Lasry, 2004; Frishammar, Flor, and Wincent, 2011). Researchers thus experiment with different methods to increase the customer's ability to comprehend and evaluate RNP concepts (e.g., Dahan and Srinivasan, 2000; Dahl and Hoeffler, 2004; Hoeffler, 2003; Van den Hende, Dahl, Schoormans, and Snelders, 2012; Zhao et al., 2012).

Transportation measures the extent to which a concept test participant can imagine an RNP and its usage. According to transportation theory (Escalas, 2007; Gerrig, 1993; Green and Brock, 2000), transportation is a consumer's ability to develop a vivid mental image of a certain situation. Consumers who are transported into a situation, such as using a new product concept (Escalas, 2004; Van den Hende and Schoormans, 2012), feel immersed in the situation and their thoughts and attention focus on it (Green and Brock, 2000; Lien and Chen, 2013). In RNP concept tests, transported individuals are able to envisage an RNP vividly and easily imagine using the product.

The aim of this article is (1) to study the effect of concept presentation in a story format, technological reflectiveness, and product experience on transportation and (2) to explore the role of transportation in improving customers’ ability to enumerate advantages and disadvantages of RNP concepts and to suggest valuable improvement ideas. These ideas are valuable for companies if they are novel, feasible, and attractive to consumers (Kristensson, Magnusson, and Matthing, 2002; Magnusson, 2009; Poetz and Schreier, 2012).

The study provides a theoretical contribution to the innovation management literature by examining the simultaneous effect of multiple drivers (i.e., a presentation format and two domain‐specific skills) on valuable improvement ideas for concept tests with RNPs (Hoeffler, 2003; Zhao et al., 2012). For RNPs, prior research has only examined either a new concept test technique (e.g., mental analogies [Dahl and Moreau 2002], narratives [Van den Hende and Schoormans, 2012], or animation [Dahan and Srinivasan, 2000]) with evaluation as the outcome variable, or a single domain‐specific skill (e.g., consumers with an emergent nature [Hoffman, Kopalle, and Novak, 2010], technologically reflective users [Schweitzer, Rau, Gassmann, and Van den Hende, 2015], or lead users [von Hippel, 1986]) with idea generation or concept development as outcome variables.

Furthermore, the study extends transportation theory (Green and Brock, 2000; Van Laer, De Ruyter, Visconti, and Wetzels, 2014) to the context of concept improvement, and is, to the best of the authors’ knowledge, the first to investigate the generation of valuable improvement ideas through the enumeration of advantages and disadvantages as an outcome of transportation (see Van Laer et al. [2014] for a meta‐analysis).

As a substantive contribution, this article provides an understanding of the role of concept presentation in a story format, technological reflectiveness, and product experience as drivers of transportation. More specifically, these drivers help practitioners design concept tests that meet the requirements of RNPs in order to obtain valuable ideas to improve such products.

## Theoretical Background

The basic idea behind concept testing is to involve consumers to elicit their point of view in order to develop products they want to buy (Moore, 2013, 1982; Piller and Ihl, 2009). In concept tests, managers present a concept and gather feedback on likes, dislikes, and improvement ideas. Concept testing includes qualitative (e.g., focus groups) and quantitative methods (e.g., surveys) to gather consumer insights for refining and optimizing the concept (Crawford and Di Benedetto, 2008; Page and Rosenbaum, 1992; Peng and Finn, 2008; Wyner, 1997).

While the value of concept tests is undisputed in respect of INPs, concept test results can be biased regarding RNPs (Hoeffler, 2003; Moore, 1982; Schoormans, Ortt, and de Bont, 1995). The core problem with RNP concept tests is that it is difficult to convey to consumers a sense of the future product, its utility, and the way of using it (Fischoff, 1991; Veryzer, 1998; Ziamou, 1999). Concepts are written descriptions (with/without complementary visuals) of a new product idea, with the basic features, technology, and customer benefits of the potential product (Dahan and Srinivasan, 2000; Foley, 2012; Ozer, 1999). Functional prototypes are often not ready for the concept test phase; the product information is incomplete and consumers cannot test out the product to experience its use. Consumers can only gauge what the final product will look like, how it will work, and how they can interact with it. To compensate for the incomplete product information and lack of actual use trials with regards to INPs, consumers infer from their usage experience with similar products (Gregan‐Paxton and John, 1997; Schoormans et al., 1995; Yamauchi and Markman, 2000). Consumers lack this prior usage experience of RNPs (Hoeffler, 2003) and have a tough time classifying the concept according to existing categories (Dahl and Moreau, 2002; Moreau, Lehmann, and Markman, 2001; Moreau, Markman, and Lehmann, 2001). Consumers may thus fail to grasp the content of RNPs, resulting in biased and potentially misleading concept test results (Knudsen, 2007; Leonard, 2002; Veryzer, 1998).

To reduce imagination difficulty, companies can employ specific concept test techniques to improve the ability of users to imagine the RNP and its future use context. Methods to facilitate consumers’ ability to grasp the content of RNP concepts and imagine using the product include virtual prototypes (e.g., Dahan and Hauser, 2002) and concept presentation in a story format. The latter is a concept description in the form of a story about a character using the new product concept (e.g., Van den Hende et al., 2012).

To avoid the problem of imagination difficulty in RNP concept tests, companies can also profit from selecting specific users who have a superior capability to understand a future RNP and its usage (Schoormans et al., 1995). Users who have domain‐specific skills in a particular area are prone to develop ideas and solutions that are connected to it (Schweitzer, Gassmann, and Rau, 2014) by intuitively drawing from their skills and abilities in this domain (Pham, Lee, and Stephen, 2012). For example, lead users are users who are ahead of an important market trend and feel that they largely profit from an innovative solution that satisfies their needs in the area of this market trend (Franke, von Hippel, and Schreier, 2006; von Hippel, 1986). They develop domain‐relevant skills in this area by tinkering and experimenting to develop a solution that meets their needs (Brockhoff, 2003; Franke and Shah, 2003; Morrison, Roberts, and Midgley, 2004; Urban and von Hippel, 1988). Two groups of users are likely to possess relevant domain‐specific skills in imagining a future RNP and its usage. First, users with high expertise on products in related product categories might find it easier to imagine the utility and usage of RNP concepts (Schoormans et al., 1995). Second, technologically reflective individuals (i.e., individuals with a tendency to think about the impact of a technological product on its users and society in general) might have developed domain‐specific skills in reflecting on the use of technologies, making it easy for them to imagine a future RNP and its usage (Schweitzer et al., 2015).

### Conceptual Framework and Hypothesis Development

#### A conceptual framework for transportation

**Figure 1:**
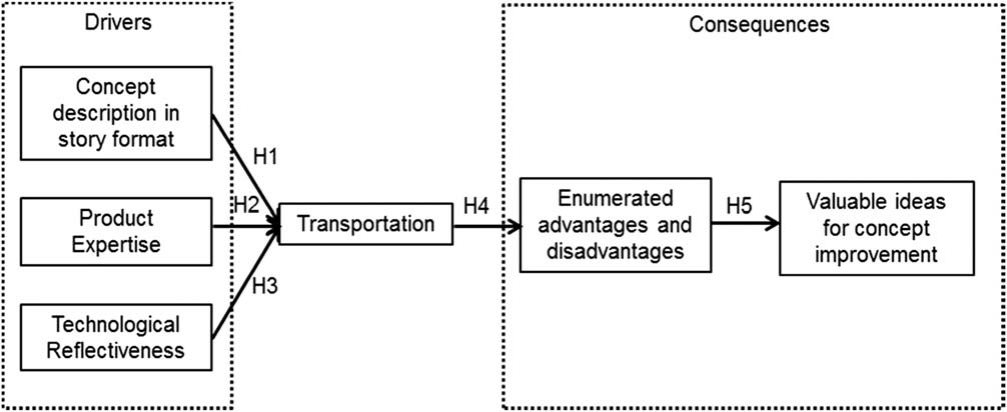
Conceptual Framework with Drivers and Consequences of Transportation in Concept Improvement Tasks Related to RNPs

Figure 1 presents the conceptual framework for transportation in concept improvement tasks related to RNPs. Transportation is conceptualized as the ability of a consumer to develop a vivid mental image of a certain situation, such as using a new product concept (Escalas, 2004; Green and Brock, 2000; Van den Hende and Schoormans, 2012). The framework establishes angles for enhancing consumers’ transportation. Furthermore, the framework explores the effect of transportation on RNP‐related concept improvement tasks in which consumers provide ideas for improving an existing concept.

The conceptual framework starts with a story format, product expertise, and technological reflectiveness as the drivers of transportation. Further, the framework comprises the ability to enumerate the advantages and the disadvantages of a concept and valuable ideas for concept improvement as consequences of transportation. Improvement ideas are valuable when they are highly novel, feasible, and benefit consumers (Kristensson and Magnusson, 2010; Poetz and Schreier, 2012).

#### Story format as driver of transportation

In product concept tests, participants usually receive a description of a concept that explains the technical characteristics of a potential new product (Dahan, Kim, Lo, Poggio, and Chan, 2011; Dahan and Srinivasan, 2000; Page and Rosenbaum, 1992). While such concept descriptions work well for INPs, they do not convey information about the product in a way that allows concept test participants to envisage RNPs well (Van den Hende and Schoormans, 2012).

A possible remedy is the description of an RNP concept in a story format. People have used storytelling for centuries to entertain others and share information (Schank, 1999; Woodside, 2010). In recent years, the effectiveness of storytelling as a means of conveying corporate information to consumers has been increasingly studied in the context of narrative advertisements (see for reviews Van Laer et al., 2014; Woodside, Sood, and Miller, 2008).

The transportation imagery model (Green and Brock, 2002; Van Laer et al., 2014) investigates the role of transportation in the context of stories (e.g., Green and Brock, 2002) or narrative advertisements (e.g., Escalas, 2004), and suggests that receivers of such narrative stimuli “generate vivid images of the story plot, such that they feel as though they are experiencing the events themselves” (Van Laer et al., 2014, p. 799).

Concept presentations in story format feature a storyline about somebody using this new product in a particular setting (Van den Hende et al., 2012). Story formats elicit transportation: The reader receives a vivid mental image of the events in the story and relates to the main character (Escalas, 2007; Gerrig, 1993). Such mental simulations can provide a surrogate product experience with RNPs to compensate for a lack of real product experience when this is not possible, for example, when the functional RNP prototypes are not yet ready (Van den Hende and Schoormans, 2012). Concept description in a story format may facilitate processing of new information and may lead to faster, and more holistic, understanding of novel information. The story format transports consumers to environments that are otherwise inaccessible and gives them the opportunity to visualize themselves in these environments. Similar to other narrative formats, concept presentation in story format can elicit transportation (Green and Brock, 2000; Van den Hende and Schoormans, 2012). The following hypothesis is thus in line with previous research:

*H1: Concept presentations in story format stimulate transportation into RNPs more than a concept presentation in a list format*.

#### Product expertise as driver of transportation

Transportation depends not only on the way the situation is presented, but also on the personal background, skills, and experience of the consumers (Van Laer et al., 2014). Domain‐specific skills are a combination of personal background, skills, and experience enabling individuals to carry out a task within a certain area (Amabile, 1996; Sawyer, 2006; Weisberg, 2006). For example, chess experts have domain‐specific skills that novices have not attained. Individuals with skills in a particular domain possess superior capabilities to solve problems in this domain. They tap their domain‐specific skills when confronted with a task that lies within this domain (Chi, Feltovich, and Glaser, 1981; Ericsson and Lehmann, 1996) and tend to develop ideas and solutions that are connected to this domain (Füller, Matzler, Hutter, and Hautz, 2012; Schweitzer et al., 2014). For example, lead users possess superior knowledge in the domain of a specific trend and often develop prototypes in this domain (Franke et al., 2006; Lettl, Hienerth, and Gemuenden, 2008; von Hippel, 1986).

For users in concept test situations, product expertise is a relevant domain‐specific skill (Schoormans et al., 1995). Product expertise is crucial for processing and understanding the information presented in a concept test. While product expertise is available in respect of INPs due to the prior knowledge of users and their personal experience, the contrary is true of RNPs (Hoeffler, 2003). Users have neither prior knowledge of the RNP nor personal experience with it. Nevertheless, some users have expertise in related product categories.

The knowledge‐transfer paradigm suggests that individuals transfer knowledge from a familiar to an unfamiliar domain (Gentner, 1989; Markman and Wisniewski, 1997). To comprehend RNP concepts, consumers map the knowledge from familiar product categories to RNPs to comprehend the latter (Gregan‐Paxton, Hibbard, Brunel, and Azar, 2002; Moreau, Lehmann, et al., 2001).

The amount of transferable information varies between consumers, depending on their knowledge base of familiar product categories (Alba and Hutchinson, 1987; Cordell, 1997). For example, in the case of the first digital camera, consumers with prior knowledge of cameras and computers could transfer this knowledge to the digital camera, while consumers with no experience in one or both categories lacked transferable knowledge.

Owing to their knowledge base, consumers with high expertise of products in related categories might find it easier to process and understand the information contained in an RNP concept description and to build a visual scenario of the product in use, thus experiencing transportation. Schoormans et al. (1995) and Peng and Finn (2010) show that consumers with product expertise provide more consistent evaluations of RNPs and more stable evaluations over time, probably through a personally induced vivid experience of the RNP. In a similar vein, Pham et al. (2012) demonstrate that consumers with product expertise are better at predicting future events within their area of expertise. The following hypothesis posits:

*H2: The higher the degree to which consumers have product experience in related product categories, the higher their transportation into RNPs*.

#### Technological reflectiveness as driver of transportation

Technological reflectiveness (i.e., the ability to “think about the impact of a technological product on its users and society in general” is a second domain‐relevant skill in the context of RNP concepts (Schweitzer et al., 2015, p. 849). Technologically reflective consumers gather their knowledge through information and use experience with technological products and through their consideration of the impact of the usage. Their engagement with technical products is not limited to their personal usage requirements; they reflect on the consequences of the usage for themselves, different social groups, and society at large. Through their reflection, these consumers explore their experiences and gain new understanding of a domain (Boud, Keogh, and Walker, 1985; Schweitzer et al., 2015). Reflection also enables these consumers to connect prior experiences with new ones, to analyze the consequences of alternate paths of action, to draw conclusions, and to act on them (Ennis, 1996).

Technologically reflective individuals have a habit of visualizing future technical products and what they can offer society. Over time, these individuals develop their capability to understand the interactions between technology and society (Schweitzer et al., 2015). By imagining and explicitly evaluating technical developments and their societal impact, these individuals may build domain‐specific skills that improve their ability to develop a vivid mental image of an RNP concept and its usage.

Since technologically reflective consumers think about technological products’ impact on different social groups, they are likely to be in a better position to imagine the use environments of an RNP for different social groups and to grasp the consequences when such groups encounter the RNP (Schweitzer et al., 2015). Technologically reflective consumers may thus be in a good position to develop a vivid mental image of an RNP and its potential usage. This is the case even when the product is merely a concept, is presented as written text, or cannot be tested as a prototype or by an advanced new concept technique that facilitates visualization. The advanced reflection skills of technologically reflective consumers lead to their personally induced transportation. The hypothesis thus postulates:

*H3: The higher the degree to which consumers are technologically reflective the higher is their transportation*.

#### Enumeration of advantages and disadvantages as outcome of transportation

Imagining the future usage of INPs is generally perceived to be an easier task than imagining this in respect of RNPs (Hoeffler, 2003). Consumers’ lack of transportation is often mentioned as a main barrier to gathering meaningful consumer information on RNPs. Knudsen (2007), for example, mentions that “the average customer may be unable to conceptualize ideas beyond the realm of his or her own experience” (pp. 117–18).

Previous research on consumer integration into NPD refers to the potential difficulties that consumers may have with contributing vital input (e.g., ideas or needs) to RNPs, due to their limited knowledge and usage experience of these (Hamel and Prahalad, 1994; Veryzer, 1998). In turn, increased transportation of RNPs increases consumers’ ability to provide such input. By facilitating the ability to fully imagine a product in action and fostering an understanding of the product concept, transportation can facilitate elaborative thoughts about the product (Block and Keller, 1997). Such thoughts explore the quality of the concept in the environment in which it is used, and include the exploration of its positive and negative aspects (i.e., elaboration of its advantages and disadvantages). Transportation is useful in the context of NPD as a vivid mental experience of a fictional product usage scenario (i.e., transportation) results in a more positive evaluation of RNPs and their perceived ease of use (Van den Hende and Schoormans, 2012). Transported consumers can easily envisage using a product, resulting in reduced adoption uncertainties and increased adoption intention (Castano, Sujan, Kacker, and Sujan, 2008).

Understanding the concept is an essential prerequisite, not only for positive evaluation and adoption, but also for considering the positive and negative aspects of a new technical concept (Veryzer, 1998). By showing consumers the full picture, transportation facilitates the generation of the advantages and the disadvantages of such a concept. This leads to the next hypothesis:

*H4: Transportation increases the consumer's ability to enumerate the advantages and the disadvantages of RNPs*.

#### Valuable ideas as results of the ability to enumerate advantages and disadvantages

Creative problem solving involves identifying an opportunity, or problem, as the first step toward seizing an opportunity or solving a problem (Isaksen, Dorval, and Treffinger, 1994; Osborn, 1953). By understanding the advantages and the disadvantages of a product, consumers have specific starting points for generating creative ideas to improve product concepts. A larger base of advantages and disadvantages provides a plurality of angles for strengthening the advantages and reducing the disadvantages. Prior research has demonstrated that a large quantity of starting points for generating new product ideas increases the quality of the ideas (Stam, de Vet, Barkema, and De Dreu, 2013; Valgeirsdottir, Onarheim, and Gabrielsen, 2015). Based on the identified advantages and disadvantages, consumers can develop ideas for strengthening the perceived advantages or reducing the perceived disadvantages (Isaksen et al., 1994; Proctor, 2013).

The ability to envision how a specific concept can on the one hand solve problems and satisfy needs, or on the other hand pose challenges to potential future customers, is important in creating RNPs that potential customers will accept.

*H5: The ability to enumerate more advantages and disadvantages of new product concepts increases the ability to generate valuable ideas for concept improvement (i.e., ideas that are novel, feasible, and beneficial to the consumer)*.

## Research Method

### Sample

The study uses 253 participants who were selected by means of a quota‐sampling procedure. Local residents living in the vicinity of the university campus were approached by a group of 27 trained research assistants, because the goal of the study was to sample ordinary users. The research assistants received a quota plan based on the age, gender, and income distribution of the population. The research assistants contacted respondents by phone and asked them to participate. Those willing to do so received an email with a link to the first part of the study (self‐administered online questionnaire) and a date for the second part of the study (on‐site concept test). As an incentive to participate, the participants received a voucher worth EUR 5 for a local supermarket chain. The sample consisted of 52.6% women (47.4% men). The median age class was 40–44 (on an answering scheme ranging from 20 to 80 in equally distributed age classes), the median net monthly income ranged between EUR 1200 and EUR 1799, while 47% of the sample had completed secondary education (53% held a college degree, or a higher postsecondary education qualification). Chi‐square tests comparing the sample with the general distribution in the population confirmed that the sample represented the general population regarding age (chi² = 4.130; *P* = .389), gender (chi² = .099; *P* = .753), and income (chi² = 7.231; *P* = .204).

### Stimuli

The study used two versions of an RNP concept description: a concept presentation in story format and a concept presentation in a nonstory, list format. The two versions were similar in terms of the number and content of the described product features, the number of times the product name was mentioned, and the description's length and elaboration. The concept presentation in story format used nonprosaic, plain language, and a classical storyline with a beginning (introduction of the main character and concept), a middle (main character performs actions with the product), and an end (main character ceases using the product and leaves the setting) (Green, 2004). The narrative elements, such as the temporal order of events and their logical interrelatedness, were removed from the story to construct the concept presentation in a list format (following a similar procedure used by Adaval and Wyer, 1998).

The key characteristics of the concept in both description formats can be summarized as follows: In line with Veryzer's (1998) definition of an RNP, the focal RNP concept was a product‐service system of an at‐home, e‐health monitor device that featured a new technology (e.g., a combination of sensors measuring the users’ blood sugar level and heart rate), offered new benefits (e.g., at‐home monitoring with direct online GP feedback), and required new behavior (e.g., the users have to take a blood sample and track their weight measurements online). E‐health solutions are among the innovations demanding considerable changes in consumption and social practices, such as increased patient responsibility and less face‐to‐face interaction with a doctor (Bechtold and Sotoudeh, 2013; Edwards‐Schachter, Matti, and Alcántara, 2012; Janssen and Moors, 2013). The e‐health stimuli in this study facilitated independent living, could automatically transfer a patient's medical data to a nursing service, allowed self‐medication based on medical analyses, and activated an alarm if the biometric data exceeded the threshold values. The chosen RNP concept required not only advanced technology, but also a functioning system of services. Furthermore, the concept description touched on issues of medical privacy (e.g., “*The Health Monitor automatically sends data to a doctor. Through online consultations, a doctor analyzes these data longitudinally and looks for changes in the key indicators to detect early evidence of dangerous diseases, such as cancer or cardiac diseases*”) and required changes in consumer behavior, such as self‐health checks instead of consulting a doctor (e.g., “*A blood sample can be taken by inserting a fingertip in a tube at the side of the Health Monitor. A thin needle that can barely be felt pricks the finger and blood drops need to be wiped onto a control strip inside the Health Monitor*”). The full texts are detailed in Appendix.

The format conditions and procedure of the two concept descriptions were pretested extensively to ensure the descriptions and questions were clear and that the timing of the tasks was correct (which is comparable to the procedure described by Kristensson and Magnusson, 2010).

### Design and Procedure

The empirical study consisted of two parts; part one took place two weeks prior to part two to minimize the carry‐over effects. Part one was a self‐administered online questionnaire, in which the participants provided information regarding their product expertise and technological reflectiveness, along with other personal data such as demographic information. For the second part of the study, the participants came to the university to participate in a concept test. The concept test manipulated two conditions of the concept description format (concept presentation in a story format vs. a concept presentation in list format). Respondents were randomly assigned to either the story format (*n* = 119) or to the list format (*n* = 134).

Part two of the study took place on site, in runs of between two and ten participants who completed the concept test individually. After reading the concept description, the participants completed a questionnaire, which included questions related to the perceived newness of the product, their comprehension of the product concept, and the transportation measure. Subsequently, the participants had to carry out two tasks. First, they were given five minutes to write down the advantages and disadvantages of the product concept (“Please enumerate the [potential] advantages and [potential] disadvantages for the consumer of using Health Monitor”), which is comparable to Hoeffler's (2003) procedure. Second, the participants had to suggest improvements to the concept. The respondents could suggest improvements in the features or uses of the Health Monitor. Similar to other on‐site ideation studies (Franke, Schreier, and Kaiser, 2010; Kristensson and Magnusson, 2010), the time for providing ideas was limited to 10 minutes. The following text introduced this task: “New or improved features/uses: Please think of ways to improve the current Health Monitor concept. Feel free to suggest any changes in the features, attributes, or uses that could improve the concept.” Furthermore, the respondents were prompted to present as many ideas as they could without allowing anything to hamper their creativity. Finally, the respondents were tested by means of the alternative use task measure of the Torrance Test of Creative Thinking to assess their general creativity (Torrance, 1990).

Individual sessions instead of group workshops were used, because prior research has questioned the efficacy of group methods and found individual creativity processes to be more effective in generating new product and service ideas (Griffin and Hauser, 1993; Paulus and Dzindolet, 1993; Schirr, 2012).

### Measures

In part one, the self‐administered online questionnaire included the seven‐item technological reflectiveness scale by Schweitzer et al. (2015) (e.g., “I enjoy thinking about ways in which future technology could change our society” with answer options ranging from 1 = “strongly disagree” to 7 = “strongly agree”; *M* = 3.90, SD = 1.66), as well as questions on product expertise. The researchers adapted the expertise scales by Sussman and Siegel (2003) and Bhattacherjee and Sanford (2006) to fit the purpose of the study (e.g., “How knowledgeable are you on using ICT products (e.g., mobile, laptop)?” with an answer scale ranging from 1 = “not knowledgeable at all” to 7 = “very knowledgeable”; *M* = 5.22, SD= 1.72). Table 1 provides a comprehensive overview of the variables used for the constructs.

**Table 1:** Results of Exploratory and Confirmatory Factor Analysis of Constructs

Constructs and Indicators	Mean	SD	ITTC	EFL	CFL
*Product Expertise (α = .95; AVE = .91; CR = .95)*					
How knowledgeable are you on using ICT products (e.g., mobile, laptop)?	5.22	1.68	.911	.977	.938
How knowledgeable are you on using the internet?	5.23	1.84	.911	.977	.971
*Technological Reflectiveness (α = .89; AVE = .53; CR = 89)*					
1. I enjoy thinking about the chances and risks a new technology might provide and harbor for society.	3.72	1.66	.739	.822	.788
2. I am very interested in studying the impact that new technical products have on society.	4.78	1.68	.626	.727	.686
3. When I hear about a new technical product, I have spontaneous ideas on how this product can be used to reduce social problems.	3.47	1.56	.600	.705	.641
4. I enjoy thinking about the impact that new technical products have on different social groups (e.g., the elderly, the young, the chronically ill).	3.72	1.62	.701	.792	.747
5. When I hear that a new technical product is on the market, I immediately reflect on the consequences this product may have for society.	3.75	1.57	.630	.731	.671
6. I enjoy thinking about ways in which future technology could change our society.	4.06	1.77	.735	.818	.789
7. I often think about how technical products could impact the autonomy and self‐determination of individuals and social groups.	3.80	1.73	.692	.785	.745
*Transportation (α=.79; AVE=.50; CR=.79)*					
1. While I was reading, I had a vivid mental image of a person using the Health Monitor in an everyday situation.	4.66	1.96	.559	.755	.696
2. While I was reading, I had a vivid image of the Health Monitor.	4.85	1.66	.686	.845	.820
3. While I was reading the text, I could easily envision what I was reading.	5.22	1.57	.633	.812	.724
4. While I was reading the text, I easily pictured a working Health Monitor.	5.08	1.63	.519	.723	.657

*α* = Cronbach's alpha; AVE = average variance extracted; CFL = factor loadings in confirmatory factor analysis; CR = construct reliability; EFL = factor loadings in exploratory factor analysis; ITTC = item‐to‐total correlations; SD = standard deviation.

The part two questionnaire that respondents answered after reading the product concept description included several measures that checked the concept descriptions again, such as the perceived newness of the concept (“How would you rate this product in terms of being unique compared to the products currently sold?” with answer options ranging from 1 = “not at all unique” to 7 = “very unique”; *M* = 5.20, SD= 1.52), and the comprehension level of the product descriptions (Hoeffler, 2003) to check whether the formulation of the stimuli was equally well understood. The respondents had to answer a four‐item transportation measure adapted from Lien and Chen (2013) (e.g., “While I was reading the text, I easily pictured a working Health Monitor,” with answer options ranging from 1 = “strongly disagree” to 7 = “strongly agree”; *M* = 4.95, SD = 1.71).

To measure the respondents’ ability to enumerate the advantages and disadvantages of RNPs, two experts, who were blind to the goals of the study, first identified the individual advantages and disadvantages from the listing provided by each respondent (e.g., through identifiers such as bullet points, semicolons, commas, or periods). Second, the experts coded nonenumeration as “0” (i.e., statements like “I don't know” or “I need to try the product before I can tell”). Third, the experts coded each advantage and disadvantage as a “1.” The advantages and disadvantages were then summed up for each respondent to reflect the total number of enumerated advantages and disadvantages. Expert disagreements about the number of arguments and coding were resolved through discussion. The experts were two research assistants trained in qualitative analysis techniques. The researchers summarized each respondent's codes to build an index of the total number of advantages and disadvantages that each enumerated.

To measure the ability to generate valuable ideas for concept improvement the study applied the procedure set out by Poetz and Schreier (2012): Two experts who were blind to the study goals first rated the novelty, feasibility, and consumer benefit of each improvement idea. These two experts were not those who coded the advantages and disadvantages; they were two practitioners with professional experience in, respectively, health care products and smart products. The three variables were measured on seven‐point rating scales. Second, the agreement among the raters was assessed with Krippendorff's alphas (Krippendorff, 2004). With values of .63 for novelty, .80 for feasibility, and .72 for consumer benefit, these interrater reliability coefficients were satisfactory (Hayes, 2005). Third, the scores of the raters were averaged regarding the three quality dimensions of each respondent's ideas. Fourth, the researchers constructed an overall quality index of the ideas that each respondent provided by calculating a three‐way interaction term (novelty × consumer benefit × feasibility) (Poetz and Schreier, 2012).

The study included general creativity, level of education, and age as control variables. To assess general creativity, the researchers used the alternative use task measure of the Torrance Test of Creative Thinking (Torrance, 1990) in the part two questionnaire. The respondents were given two minutes to list as many different uses for a common brick as they could think of (Torrance, 1990). To use this measure in the analyses, the number of alternative uses that each respondent generated in this task was counted (*M* = 4.75; SD = 2.74), with high numbers representing more creative individuals. The respondents provided information on their level of education (five classes from 1 = elementary school, 2= middle school, 3= vocational school, 4 = high school diploma, 5 = university degree) and age (measured in five‐year age classes ranging from “20–24” to 75–80”) in part one of the self‐administered online questionnaire.

## Results

### Stimuli Control

An ANOVA analysis with SPSS was utilized to check the stimuli. There were no differences between the comprehension levels of the story and list formats of the RNP (story format mean = 1.92; bulleted list mean = 1.78; *F*(1, 251) = 1.09; *P* = .30). This suggests that the story and list format stimuli were equally understandable. The check for newness showed that the participants rated the Health Monitor as rather radical (*M* = 5.59; SD = 1.30).

### Reliability and Validity Measures

The SPSS‐based conventional methods of coefficient alpha, item‐to‐total correlations, and exploratory factor analysis (Churchill, 1979) served as a first reliability and validity test for the conceptual model's constructs. Each individual factor also proved reliable in the more advanced confirmatory factor analysis (Bagozzi and Baumgartner, 1994; Byrne, 2011) using Amos 23 (IBM, Zurich, Switzerland). As shown in Table 1, all the indicators had item‐to‐total correlations (ITTCs) greater than the recommended .4; factor loadings and the coefficients of all the indicators were significant (i.e., >1.96). The composite reliability of all constructs was above the .70 threshold, and the constructs met the required .50 threshold for the average variance extracted (Hair, Black, Babin, and Anderson, 2013).

Further, the Fornell–Larcker criterion tested for discriminant validity (Fornell and Larcker, 1981). In Table 2, the diagonal elements representing the square roots of the average variance extracted (AVE) were greater than the off‐diagonal elements. Thus, the constructs in this study complied with discriminant validity.

**Table 2:** Descriptive Statistics, Correlations, and Square Root of AVE of the Constructs in the Empirical Model

		Mean	SD	1	2	3	4	5	6	7	8
1	Transportation	5.95	1.34	(.704)							
2	Description format	0.47	0.50	.166**							
3.	Product expertise	5.22	1.72	.345**	.020	(.955)					
4	Technological reflectiveness	3.90	1.27	.249**	−.107	.231**	(.726)				
5	Advantages/disadvantages	5.83	3.15	.278**	.014	.468**	.282**				
6	Valuable ideas for concept improvement	22.29	15.75	.212**	.038	.374**	.205**	.457**			
7	Age	5.57	3.43	−.272**	.004	−.681**	−.118	−.448**	−.363**		
8	Creativity	4.75	2.74	.118	.005	.213**	.288**	.451**	.366**	−.198**	
9	Education	3.26	1.28	.232**	.059	.517**	.137*	.415**	.367**	−.449**	.203**

*p < .05; **p < .01; SD = standard deviation; square root of average variance extracted (AVE) is shown on diagonal in parentheses (where appropriate).

### Overall Model Fit

Table 2 shows the descriptive statistics of the measures used to test the hypotheses. The hypotheses were tested with a structural equation modeling (SEM) approach, using standardized variables as the variables had differing scales (Mahr, Lievens, and Blazevic, 2014). The absolute (goodness of fit index [GFI]; adjusted goodness of fit index [AGFI]) and incremental fit index (Tucker‐Lewis coefficient [TLI]; comparative fit index [CFI]) along with the standardized root mean square residual (SRMR) and the root mean square error of approximation (RMSEA) were calculated. The obtained values (X²/df = 1.209; GFI = .990; AGFI = .953; CFI = .996; TLI = .986; SRMR = .026; RMSEA = .029) are well within the recommended bounds (Hair et al., 2013; Hu and Bentler, 1998). Furthermore, the normed chi‐square measure showed parsimonious fit (*p* = .279) (Hair et al., 2013). Hence, the data fit the model well, thus allowing for an interpretation of the results.

### Main Hypotheses Testing

**Figure 2:**
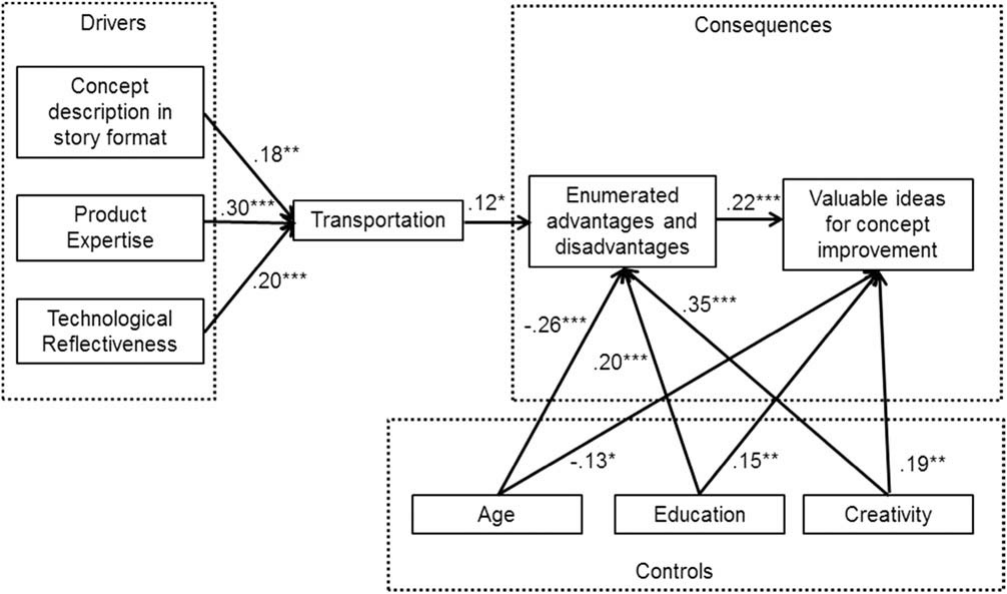
Empirical Model of Transportation in Concept Improvement Tasks Related to RNPs

The path coefficients of the model are presented in Figure 2. H1 to H3 concern the drivers of transportation. The data (*β* = .181, *p* = .002) supported H1, which postulates that the concept description in story format increases transportation (i.e., a consumer's ability to develop a vivid mental image of a concept). H2, which states that product expertise has a positive impact on transportation, also found empirical support in this full model. The impact is positive and significant (*β* = .295, *p* < .001). Moreover, technological reflectiveness significantly increased transportation (*β* = .200, *P* < .001), thus supporting H3.

H4 and H5 concern the consequences of transportation. In line with H4, transportation showed a significant and positive effect (*β* = .121, *p* < .019) on the ability of consumers to enumerate the advantages and the disadvantages of the RNP. Further, their ability to enumerate the advantages and the disadvantages of the RNP increased their ability to generate valuable ideas for concept improvement (*β* = .221, *p* < .001). These results support H5.

The controls also had a significant effect on the ability of consumers to enumerate the advantages and the disadvantages (creativity [*β* = .345, *p* < .001], age [*β* = −.257, *p* < .001] education [*β* = .202, *p* < .001]), and on their ability to generate valuable ideas for concept improvement (creativity [*β* = .187, *p* < .001], age [*β* = −.137, *p* = .020], education [*β* = .154, *p* = .008]).

To better understand the role of consumers’ ability to enumerate the advantages and the disadvantages regarding their ability to provide valuable ideas for concept improvement, the researchers looked into alternative models based on the ratios of advantages and disadvantages. A first model used a difference measure (quantity of advantages minus quantity of disadvantages) instead of the mere enumeration measure of the advantages and the disadvantages. The researchers thus endeavored to determine whether transportation provokes more positive or more negative arguments about the concept. Based on transportation literature, transportation triggers more positive evaluations (Van Laer et al., 2014) and this should lead to more positive than negative arguments. More positive arguments could make finding improvement ideas difficult; it might be easier to suggest ideas to reduce the disadvantages of concepts than to improve concepts that already exhibit strong advantages. The model showed that transportation has a significant and positive effect on the difference measure (*β* = .345, *p* < .001), but this has no effect on the ability to provide valuable ideas for concept improvement (*β* = −.040, *p* = .456).

In a second model, the absolute difference value of the enumerated advantages and disadvantages (absolute value of quantity of advantages minus quantity of disadvantages) replaced the mere enumeration measure of the advantages and disadvantages in the core model. The aim of the second model was to understand whether differences in the proportion of arguments, irrespective of their direction, led to an improved ability to provide valuable ideas for concept improvement. The model results showed that neither the link between transportation and absolute value (*β* = .063, *p* = .294) nor between absolute value and the ability to provide valuable ideas for concept improvement (*β* = −.020; *p* = .715) was significant.

The investigation of the two alternative models substantiates the relevance of a broad base of arguments about a new concept as a basis for suggesting concept improvements. The direction of the arguments does not play a major role in this context.

Further, a model with a direct link between transportation and valuable ideas for concept improvement found a strong and positive impact of transportation on valuable ideas for concept improvement (*β* = .213, *p* < .001). As a next step, the mediating effect of enumerated advantages and disadvantages on the relationship between transportation and valuable ideas for concept improvement were investigated applying the bootstrapping methodology described by Preacher and Hayes (2008) and Efron and Tibshirani (1993). Using the Preacher and Hayes (2008) approach, bootstrapping showed that narrative transportation has a significant indirect effect on generating valuable improvement suggestions (two‐tailed significance of bootstrap standard error for indirect effects: *p* < .001 with a point value of .136 and a 95% bias‐corrected confidence interval [BCaCI] of .096 to .194). Also, Sobel's (1982) *z*‐test (*z* = 3.264, *P* = .001) indicated significant mediation. This mediation effect further substantiated the model in Figure 2 and provides empirical support for the hypotheses.

## Discussion and Conclusion

This article demonstrated that three drivers facilitated participants’ transportation in a concept test for an RNP: participants’ degree of technological reflectiveness and product expertise, and a concept presentation in a story format. Such vivid examination of the RNP gave the participants a virtual experience of the RNP concept, enabling them to envisage the potential advantages and disadvantages of using it. Based on their understanding of the pros and cons of the concept usage, the participants could create ideas to improve the product concept that were valuable in terms of their novelty, feasibility, and benefit for consumers.

This study contributes to transportation theory in two ways. First, it showed the positive effect of transportation on valuable ideas that improve RNP concepts. Prior transportation research has mainly examined the persuasive and affective effects of transportation (Van Laer et al., 2014), such as critical narrative thought (Chang, 2009), affection (Escalas, 2004), persuasion (Green and Brock, 2000), and behavioral intent (Schlosser, 2003). In the context of RNP concept testing, transportation research has focused only on affection and ease of use outcomes (Van den Hende and Schoormans, 2012). Second, this study elucidated the mechanism underlying the effect of transportation on valuable suggestions for concept improvement: transportation increases the ability to enumerate advantages and disadvantages, which helps in providing valuable suggestions for improvement.

The article also contributes to the innovation management literature. First, it contributes to the literature that advocates the involvement of regular consumers in the NPD process. Prior research on consumer involvement has focused on noncomplex product categories (i.e., low knowledge required to understand how existing products work and how they can be modified [Lüthje, Herstatt, and von Hippel, 2005; Poetz and Schreier, 2012]), such as T‐shirts and granola (Schreier, Fuchs, and Dahl, 2012) or baby products (Poetz and Schreier, 2012). This article demonstrated that ordinary consumers have the ability to contribute to the development of new products in complex product categories as well.

Second, the present research contributes to concept test research by showing the simultaneous effects of three drivers on valuable ideas for improvement: one new concept test technique (a concept description in a story format) and two domain‐specific skills (product expertise in related product categories and technological reflectiveness). Prior research has examined the effects of single drivers on different concept test outcomes (e.g., attitude toward the RNP or number of generated ideas). More specifically, prior research has examined either a new technique (e.g., mental analogies [Dahl and Moreau, 2002], narratives [Van den Hende and Schoormans, 2012], or animation [Dahan and Srinivasan, 2000]) with evaluation as the outcome variable. Or, prior research has examined the effect of a single domain‐specific skill (e.g., consumers with an emergent nature [Hoffman et al., 2010], technologically reflective users [Schweitzer et al., 2015], or lead users [von Hippel, 1986]) with more elaborated idea generation as outcome variables.

Concept descriptions can include many different elements, such as consumer insights, benefits, reasons to believe, or contextual information, yet a concept description in story format goes beyond a typical concept description. A story features a main character, product use, outcomes of the use (i.e., product benefits), and the location of use (i.e., contextual information), but, most importantly, it has a logical interrelated sequence of these elements, which facilitates narrative transportation (Adaval and Wyer, 1998).

Prior research shows that dissociation from the main character in a concept test story inhibits transportation (Van den Hende et al., 2012). Extending this line of thought, any dissociation might inhibit transportation. Though price was not mentioned in the RNP concept description, price inferences might have been present. A very high anticipated price for an RNP might have limited ordinary consumers’ transportation levels, because they feel it is a “not for me” product. However, this would have led the respondents to list more disadvantages relative to advantages in the non‐story format, yet this was not the case.

The content of a story influences the perception of the reader. Transportation theory maintains that when transported into a narrative, consumers’ attitudes and preferences change in the direction of the story content and they can become less aware of real‐world facts that contradict assertions made in the narrative (Green and Brock, 2000). Using the story format for concept research would warrant thinking about the content of the story, as it steers the outcomes. The content can influence the types of advantages and disadvantages that consumers perceive. As these advantages and disadvantages form the basis of idea generation, the content can ultimately also influence the types of ideas that are generated. As such, the story format can potentially limit the breadth of the feedback to the context of the narrative vis‐à‐vis relatively more open‐ended feedback. On the other hand, it might increase the depth of feedback on a specific context through more intense immersion into this context.

### Managerial Implications

Understanding consumer involvement in the NPD process of RNPs is important as firms focusing on developing RNPs are often more successful than those concentrating on INPs (Markham and Lee, 2013). The present article focused on a technologically complex RNP with a potentially high societal impact. When seeking input from consumers on such a product during the concept development phase, innovation managers can benefit from eliciting transportation. This article offers three means to elicit transportation: a story format to describe the RNP concept, selection of consumers with high product expertise in related product categories, and selection of technologically reflective consumers.

Selecting consumers with high technological reflectiveness or product expertise demands screening for concept test participants along these characteristics. Some argue that such screening can be burdensome and resource‐intensive (Belz and Baumbach, 2010; Peng and Finn, 2010), while others consider that online selection provides opportunities (Füller and Matzler, 2007). For the story format, a classical story told in plain language suffices to elicit transportation. The length depends on the number of possible uses of the product that need to be tested, though multiple stories with single uses could also be considered.

The transportation that concept test participants experienced helped them to provide ideas for concept improvement. This was because of their better understanding of the pros and cons of the concept usage, which were made explicit by letting the participants enumerate the advantages and the disadvantages of the RNP. Such “pros and cons” thought listings are common practice in concept testing, as they also allow validating the intended benefits of the RNP.

Stories for RNP concept testing offer numerous opportunities for innovation managers. For example, prior research shows that concept tests in story formats serve as surrogates for a prototype demonstration for attitude and ease of use estimations (Van den Hende and Schoormans, 2012). Therefore, the story format could allow managers to explore multiple concepts further, before expensive prototypes are developed. Different potential future usage scenarios, such as ones developed in scenario workshops (Rau, Schweitzer, and Gassmann, 2014; Wack, 1985), could be tested in concept tests. In the former, participants could develop different scenarios for the potential future usage of an RNP, while in the latter, consumers could receive different concept descriptions in a story format, each based on one of the developed scenarios.

### Limitations and Suggestions for Further Research

The various limitations of this study offer opportunities for further research. First, the focal product was an e‐health device and the applicability of the findings to other product categories may be limited. The product was health‐related and its usage might have a high societal impact. Technologically reflective individuals might have strong transportation abilities when technological solutions are strongly linked to societal issues, but might not have these abilities when it comes to other technological products. Thus, other product categories should be examined to demonstrate the general role that technologically reflective consumers play in concept tests.

Second, this article demonstrated the positive effect of transportation on generating ideas to improve an RNP concept. However, this is only one kind of consumer involvement in the NPD process. Another consumer involvement activity is idea generation (Mahr et al., 2014), either through the consumer's own initiative or challenges (Füller and Matzler, 2007). Further research on transportation could explore this phase of the NPD process. Stimuli in story format narratives for this phase, however, are unlikely to include a product yet, and therefore scenarios of the future (Wade, 2012) could be used as stimuli in story format.

Third, respondents in the empirical study had a time limit of five minutes to enumerate the advantages and the disadvantages of the RNP concept, and 10 minutes to provide ideas for concept improvement. Time limits are general practice in concept test settings and the set time limit was comparable to time limits in other studies (e.g., Kristensson and Magnusson, 2010). However, such time pressure might have different effects on different people: While it may inhibit some people's ability to provide creative input, it might boost the ability of others (Baer and Oldham, 2006; Moreau and Dahl, 2005; Sheremata, 2000; Zhang, Zhang, and Song, 2015). To extend the validity of this study's findings to different settings, future studies could try to replicate the results in settings without a time limit.

Fourth, the measurement of the concept refinement solutions included an indirect market acceptance measure in the form of what experts view as the perceived consumer benefit. Therefore, a longitudinal study could encompass evaluation of the generated ideas followed by consumer evaluation of the final product.

To conclude, the empirical study combined online research to measure the participants’ degrees of technological reflectiveness and product expertise, and offline site visits for the concept test. However, the latter stage could also have been done online, though in that case it would perhaps have been less controlled. As such, the procedures and means described in this article (i.e., concept descriptions in story format as well as selection of participants) provide feasible opportunities for managers to get valuable input from consumers on RNP concepts.

## Concept Presentation in Story Format

Please read the following new product concept thoroughly and evaluate it afterwards by answering the questions below.

### Health Monitor

Health Monitor is a device that records eating habits, work‐out data, and Ann's health condition through blood measurements with various internal and external sensors. Ann is planning to lose 10 pounds within the next two months in a healthy way through exercise, healthy food intake, and regular online medical check‐ups by her doctor.

Ann's week starts with her Monday exercises, during which she measures her calorie consumption with Health Monitor's external chest strap sensor. When she gets home, she puts the strap next to the Health Monitor on the table in her living room and the exercise data automatically transfer to the Health Monitor. When she starts her daily routine of interactions with the Health Monitor, her daily food intake can be manually entered into the Health Monitor. She can browse through the suggestions on the Health Monitor's screen about groceries and recipes to help her reach her dietary targets healthily and sustainably. Next, she straps on the blood pressure wristband, and waits a moment for the blood pressure data to be measured and wirelessly transferred to the Health Monitor. Ann can also take a blood sample by inserting her fingertip into a tube at the side of the Health Monitor. She is pricked by a thin needle that she barely feels and wipes the blood drops onto a control strip inside the Health Monitor. This way, the Health Monitor records her cholesterol levels, blood sugar, and nutrient balance. On a weekly basis, the Health Monitor automatically sends Ann's data to her doctor. Through online consultations, the doctor analyzes this data longitudinally and looks for changes in the key indicators to detect early evidence of dangerous diseases, such as cancer or cardiac diseases.

On Friday, Ann enters her food intake and weight measurement, and the Health Monitor records her blood values in the usual way. Next, she studies the results of the weekly analysis that she received from her doctor on the screen of the Health Monitor. She ponders the few options that the device suggests for lowering her cholesterol values, which have been above average over the last two weeks. Thereafter, Ann plugs the Health Monitor into her laptop, logs into the Health Monitor website, presses the button for groceries, and receives information on the prices and sales of the suggested groceries at the supermarkets in her vicinity. Ann selects the grocery items she wants, and, instead of ordering them online as she sometimes does, she prints her selection. She wants to meet a friend at a coffee shop in half an hour and plans to buy the selected items on her way home. She grabs the list and her phone and leaves the house.

## Concept Presentation in List Format

Please read the following new product concept thoroughly and evaluate it afterwards by answering the questions below.

### Health Monitor

- Health Monitor is a device that records eating habits, work‐out data, and health condition through blood measurements with various internal and external sensors.
- It helps to achieve a set weight dietary goal in a healthy way through exercise, healthy food, and regular online medical check‐ups by a doctor.
- Health Monitor's external chest strap sensor can measure calorie consumption when exercising.
- At home, the strap needs to be next to the Health Monitor that can be on the living room table. As soon as the strap is placed near the Health Monitor, exercise data are automatically transferred to the Health Monitor.
- The daily food intake can be entered manually into the Health Monitor as part of a daily routine of interactions.
- The Health Monitor's screen shows suggestions about groceries and recipes to reach dietary targets healthily and sustainably way through which the user can browse.
- With the blood pressure wristband, it takes a moment to measure the blood pressure data that are wirelessly transferred to the Health Monitor.
- A blood sample can be taken by inserting a fingertip into a tube at the side of the Health Monitor. A thin needle that can barely be felt pricks the finger and blood drops need to be wiped onto a control strip inside the Health Monitor.
- The Health Monitor records cholesterol levels, the blood sugar, and the nutrient balance.
- On a weekly basis, the Health Monitor automatically sends data to a doctor. Through online consultations, a doctor analyzes these data longitudinally and looks for changes in the key indicators to detect early evidence of dangerous diseases, such as cancer or cardiac diseases.
- Food intake and weight measurements can be entered, and the Health Monitor also records blood values.
- The results of the weekly analysis by a doctor can be studied on the screen of the Health Monitor.
- The device suggests options to respond to medical data such as high cholesterol values.
- Information can be provided on the prices and sale of the suggested groceries in the supermarkets in the vicinity. To do so, the Health Monitor needs to be plugged into a laptop, its website needs to be accessed, and the groceries button needs to be pressed. The website provides the possibility to order selected items online, or to print the selection. The latter is necessary if, for example, the groceries can be bought on the way home after a coffee appointment with a friend and the printed list is needed.
